# Fabrication of Janus Supraparticles by Induced Phase Separation by Gravity

**DOI:** 10.1021/acsnano.5c20500

**Published:** 2026-04-15

**Authors:** Sayanth Ramachandran, Michael Kappl, Marcel Sperling, Michael Gradzielski, Hans-Jürgen Butt

**Affiliations:** † Department of Physics at Interfaces, 28308 Max-Planck Institute for Polymer Research, 55128 Mainz, Germany; ‡ Stranski-Laboratorium für Physikalische und Theoretische Chemie, 26524 Technische Universität Berlin, Strasse des 17. Juni 124, Berlin 10623, Germany

**Keywords:** supraparticle, Janus particle, evaporation, nanoparticle assembly, superamphiphobic surface, sedimentation

## Abstract

Janus supraparticles, which are anisotropic colloidal assemblies with phase-separated domains, have attracted significant interest for their structural and functional versatility. However, fabricating Janus supraparticles is challenging because it requires precise control over the spatial arrangement of different micro- or nanoparticles within a single assembly. While phase separation of a nanoparticle mixture with an external gradient field is studied with magnetic particles, gravity is unexplored as a potential external field. In this work, we demonstrate the synthesis of Janus supraparticles by evaporation of binary suspensions on a superamphiphobic substrate. We find that combining particles of similar size but different densities, as well as those of the same material with varying sizes, leads to an effective internal phase separation. These results highlight the potential of leveraging sedimentation as a particularly simple yet robust strategy to induce spatial heterogeneity during supraparticle formation.

## Graphical abstract

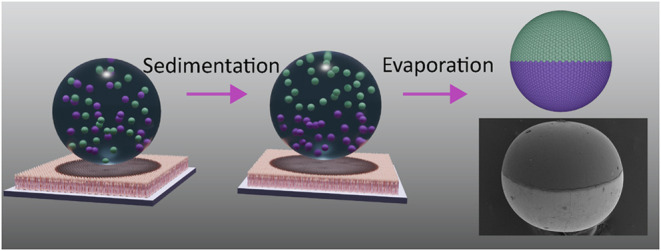

## Introduction

Supraparticles (SPs) are hierarchical structures formed from colloidal nanoparticles (NPs) as building blocks, through self-assembly driven by internal forces or external confinement.^1–3^ Their structure, composition, and properties can be tuned by selecting and arranging the constituent NPs, which may vary in size, shape, and chemistry.^4,5^ As a result, SPs hold emergent functionalities arising from the interactions between different types of building blocks apart from the functionalities of individual NPs,^2,6^ while reducing the hazards caused by the high mobility of nanoscale colloids. These characteristics make them promising for diverse applications, including catalysis,^4,7,8^ photonics,^9,10^ drug encapsulation and delivery,^11,12^ energy production and storage materials,^13^ gas adsorption and sensing.^14,15^

Extensive research has been carried out on single-component systems to uncover the fundamental mechanisms underlying SP formation.^5,6,16–18^ To enhance both structural and functional complexity, efforts have been made to develop multicomponent SPs incorporating NPs with different functionalities.^19–22^ Among such systems, Janus particles stand out due to their intrinsic structural asymmetry. First introduced by de Gennes in his 1991 Nobel lecture “Soft Matter”,^23^ Janus particles are named after the Roman god Janus, having two faces. This asymmetry can impart two chemically or physically distinct faces, enabling the integration of contrasting properties and directional behavior within a single particle. This makes them promising for diverse applications, such as sensors, catalysts, foam and emulsion stabilizers, carrier and absorber materials, and magnetic/electronic switchable composites.^24–28^

Different techniques have been developed to fabricate SPs from droplet-based templates.^1,29,30^ Typically used templates for wet self-assembly, where confinement is enforced by the liquid–liquid interface, are emulsions^9,31–33^ or microfluidically generated droplets.^34,35^ While effective, these methods often involve additional processing of liquids that complicate disposal and offer limited control over the final SP morphology. Dry self-assembly methods, in contrast, rely on solvent evaporation from the liquid–air interface that eliminates the need for additional liquids, simplifying the recovery of assembled structures. Common dry self-assembly techniques include spray drying^36–39^ and Leidenfrost levitation,^40,41^ which operate at higher temperatures over short time scales, making them less suitable for understanding structural formation. In this context, the superamphiphobic surface-assisted method provides a more accessible alternative. Here, a suspension droplet is dried on a super liquid-repellent surface characterized by a contact angle greater than 150° that helps liquid drops to maintain spherical shape.^42,43^ The solvent evaporation is horizontally symmetric. In addition, the porous nature of the substrate further facilitates solvent removal through the surface, ultimately leaving the spherical SP behind.^4,44–46^ This process can be readily extended to parallel droplet-based fabrication approaches through controlled droplet generation methods, such as inkjet printing or droplet arrays. Here, evaporation-driven assembly occurs in parallel with precise control of the droplet volume and placement. A practical limitation arises from the use of highly liquid-repellent substrates, which require sufficient spacing between droplets to prevent coalescence during deposition. Nevertheless, the parallel deposition of large droplet arrays provides a feasible pathway toward increased production. This technique also allows the processing of nonaqueous suspensions. The slower kinetics in this approach make it particularly well-suited for investigating the dynamics of structure formation in real-time.

Various methods have been developed to synthesize Janus particles.^25,47,48^ However, only limited studies have explored the assembly of binary NP mixtures into a single, asymmetrically structured Janus Supraparticle (JSP).^39,49–52^ A key challenge in forming JSPs is inducing coordinated motion and phase separation among different NP species. This has been addressed in part by applying external gradient fields, such as magnetic fields, which require the use of magnetic components.^49,53^ However, the potential of gravity as a driving force has remained unexplored. The influence of gravity on colloidal suspensions is an interplay between buoyancy and diffusion.^54^ While NP sedimentation has been extensively studied in bulk systems,^55,56^ its behavior within a spherical droplet is not yet fully understood. Additionally, integrating sedimentation inside a droplet on a superamphiphobic surface may offer a straightforward, one-step approach to producing JSPs.

In this study, we investigated the role of gravity in the sedimentation of NPs within an evaporating spherical droplet. We fabricated JSPs from binary mixtures of spherical NPs with different buoyant masses on a superamphiphobic surface (Figure 1). This approach is material-independent; thus, functional behavior can be introduced by selecting primary NPs with desired properties. Mixtures comprising polystyrene (PS) and silica (SiO_2_) NPs of similar size, and PS–PS mixtures with distinct particle sizes, exhibit clear phase separation within the SPs. The key factor in achieving this separation is the use of a high relative humidity (RH), which slows the evaporation rate and allows sufficient time for the complete sedimentation of the heavier components. This one-step assembly strategy simplifies the fabrication process. It also enables control over the sharpness of the phase boundary, providing a tunable platform for constructing structurally anisotropic JSPs.

**Figure 1:**
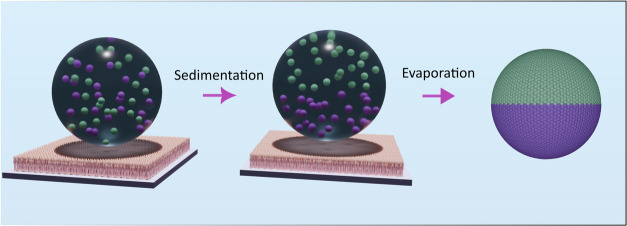
Schematic illustration of Janus supraparticle formation from sedimentation mediated by droplet evaporation on a superamphiphobic surface.

## Results and Discussion

### Fabrication of Janus Supraparticles

We fabricate SPs by placing a droplet of a suspension of particles on a superamphiphobic surface. The surface is prepared by hydrolysis of silane (Figures 2a and S1).^57,58^ The resulting substrate exhibits excellent liquid repellency, with a static contact angle of ≈157° for water and ≈148° for hexadecane droplets (both 6 μL) (Figure 2b,c). Such liquid repellency ensures the formation of nearly spherical droplets of NP suspensions, provided that the drop diameter is below the capillary length $\kappa =\sqrt{\gamma /\rho g}\approx 2.7\;\mathrm{mm}$ . Here, γ is the surface tension of the suspension, ρ is its density, and *g* is the acceleration of gravity.

**Figure 2:**
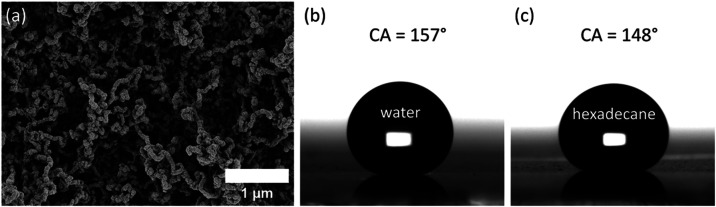
Nanofilament-based superamphiphobic surface for spherical droplets. (a) SEM image of the nanofilaments forming the superamphiphobic surface. (b) Side-view images of 6 μL water and (c) hexadecane droplets on the superamphiphobic surface.

At equilibrium, in the presence of gravity, the suspension of particles develops an inhomogeneous density profile along the vertical direction. Piazza et al have attempted to explain this phenomenon by accounting for the thermal fluctuations of particles and the buoyant force during sedimentation.^54^ The volume fraction of the NPs at equilibrium at a height *z* from a reference point is given by

1 $$\phi \left(z\right)=\phi \left(0\right)e^{-z/l_{g}}$$

This introduces *l*_g_ = *k*_B_ *T*/m*g, the sedimentation length of the particles. Here, *k*_B_ is the Boltzmann constant and *T* is the temperature. *l*_g_ is assumed to be much larger than the particle diameter for stable suspensions without sedimentation. m* is the buoyant mass and is given by

2 $$m^{*}=\Delta \rho V$$

Here, Δρ is the density difference between the particle and the medium, and *V* is the particle volume. This demonstrates that the density and size of the particles are variables contributing to the gravitational length and thus to the sedimentation profile of the suspension. To estimate whether sedimentation will effectively occur before the droplet dries, we define a sedimentation time τ_s_ as the time for a particle to settle a distance equal to the initial droplet radius *R*. Using Stokes’ law, the sedimentation velocity, *v*, is

3 $$v=\frac{2r^{2}g\Delta \rho }{9\eta }$$

Here *r* is the particle radius and η is the viscosity of the medium. From this, the sedimentation time is estimated as

4 $$\tau_{s}=\frac{9\eta R}{2r^{2}g\Delta \rho }$$

The estimated τ_s_ provides an approximate lower bound for the time required for sedimentation under gravity, without Brownian motion and other hydrodynamic effects. While actual settling times could be longer, this estimate becomes more accurate for larger or denser particles.

In our studies, we prepared a binary mixture of suspensions with NPs of different *l*_g_ as primary building blocks of JSPs. Particles having *l*_g_ in the range of their diameter are considered to be sedimentation-dominated, and particles with *l*_g_ much larger than particle diameter are diffusion-dominated. We calculated τ_s_ for NPs inside a 2 μL droplet by inserting 700 μm as the droplet radius. Estimated τ_s_ demonstrate that sedimentation-dominated and diffusion-dominated particles behave consistently within the experimental time scales (Table 1). We chose PS NPs with a mean diameter of 608 nm (density = 1060 kg/m^3^, labeled with the fluorescent dye Nile red), and SiO_2_ NPs with a mean diameter of 622 nm (density = 1880 kg/m^3^) as the primary particles, where PS is diffusion-dominated and SiO_2_ is sedimentation-dominated in this size range. We dispensed 2 μL of this binary mixture onto the superamphiphobic surface. The drying speed was controlled by varying RH, which is a significant factor in sedimentation. The suspension dried with a constant contact angle, without contact line pinning.

**Table 1:** Sedimentation Length (*l*_g_) and Sedimentation Time (*τ*_s_) for Polystyrene and Silica Nanoparticles Inside the Droplet with a Radius of 700 μm

nanoparticle	diameter (nm)	density (kg/m^3^)	*l*_g_ (μm)	τ_s_ (h)
polystyrene	608	1060	58.9	15.4
polystyrene	1793	1080	1.72	1.3
silica	152	1890	253.8	16.6
silica	622	1880	3.74	1.0

The evaporation-driven self-assembly of particles can be described as an advection-diffusion process in confinement. The evaporating air–solvent interface creates an effective advection that can reduce particle sedimentation.^59^ Along with that, droplets of a few microliters can dry before any effective sedimentation. Both of these issues are overcome by decreasing the drying speed. To ensure complete sedimentation before complete evaporation, we conduct all our experiments at high humidity in a humidity chamber with RH in the range of 95%–99% (at 22°*C*).

The droplet dried within 12 h. At high RH (>95%), the drying time is highly sensitive to small variations in humidity. Thus, the exact drying time can vary on the order of an hour between experiments. We always dried multiple droplets simultaneously during each experiment, allowing a comparison of evaporation behavior at different positions within the chamber. The resulting SPs showed consistent morphology and internal structure across the sample set, indicating uniformity in the humidity inside the chamber. We observed the reduction in the droplet volume with a side-view camera (Blackfly S BFS-U3–50S5C). We illuminated the droplet from the same direction as the camera using a lamp with an optical fiber waveguide to observe the phase separation during evaporation. This observation suggests that flow-driven particle movement is prominent only in the initial stage after droplet deposition. As drying proceeds, the evaporation rate decreases, and sedimentation becomes increasingly important (Video S1, playback speed 5× relative to real time). Of course, this approach is limited to qualitative characterization; for a quantitative analysis, particle tracking would be needed, which would become difficult for the dense suspensions at the later stage of evaporation.

For a dilute suspension, the configurational entropy is maximized by a homogeneous distribution of the particles within the liquid. In this situation, entropy opposes sedimentation, leading to the density distribution described by eq 1. In highly concentrated suspensions (the latter stage of drying), the configurational entropy per particle gets reduced, and the translational entropy becomes more important. This leads to the situation that entropy favors a higher ordered configuration, as the crystalline arrangement of the particles leads to higher packing, leaving more space for particle movement and thus a higher translational entropy. Thus, at the later stage of sedimentation, entropy acts as a driving force for crystallization of the particles and compaction of the structure.

We observed structural colors on the surface of the droplet during evaporation and later on the SP that are indicative of the formation of colloidal crystals by PS and SiO_2_ NPs (Figure 3a). Once SiO_2_ settles, it crystallizes at the bottom, giving rise to structural color. Subsequently, PS crystallization initiates at the SiO_2_–PS boundary and propagates upward through the dispersed PS domain, eventually leading to color across the entire PS region. The shrinking air–water interface leads to upconcentration of the particles close to the interface, leading to spherical shell formation. This shell is dense but not yet arrested due to very high humidity and low evaporation rate, forming a colloidal crystal where particles are tightly packed but not yet touching. This is reflected by homogeneous and symmetric shrinkage of the drying droplet. The optical response indicates ordered packing in both components, enabled by their refractive index contrast relative to water (*n*_PS_ ≈ 1.59, *n*_SiO_2__ ≈ 1.45, *n*_water_ ≈ 1.33). The size mismatch between the PS and SiO_2_ particles is small enough that they might cocrystallize. However, we observe almost complete phase separation and a sharp boundary between the two phases. This could hint that crystallization acts as an additional driving force for the removal of particles of the counter species from the crystal lattice. The colorful reflections also indicate that both hemispheres are monodisperse and the phase separation is pronounced.

**Figure 3:**
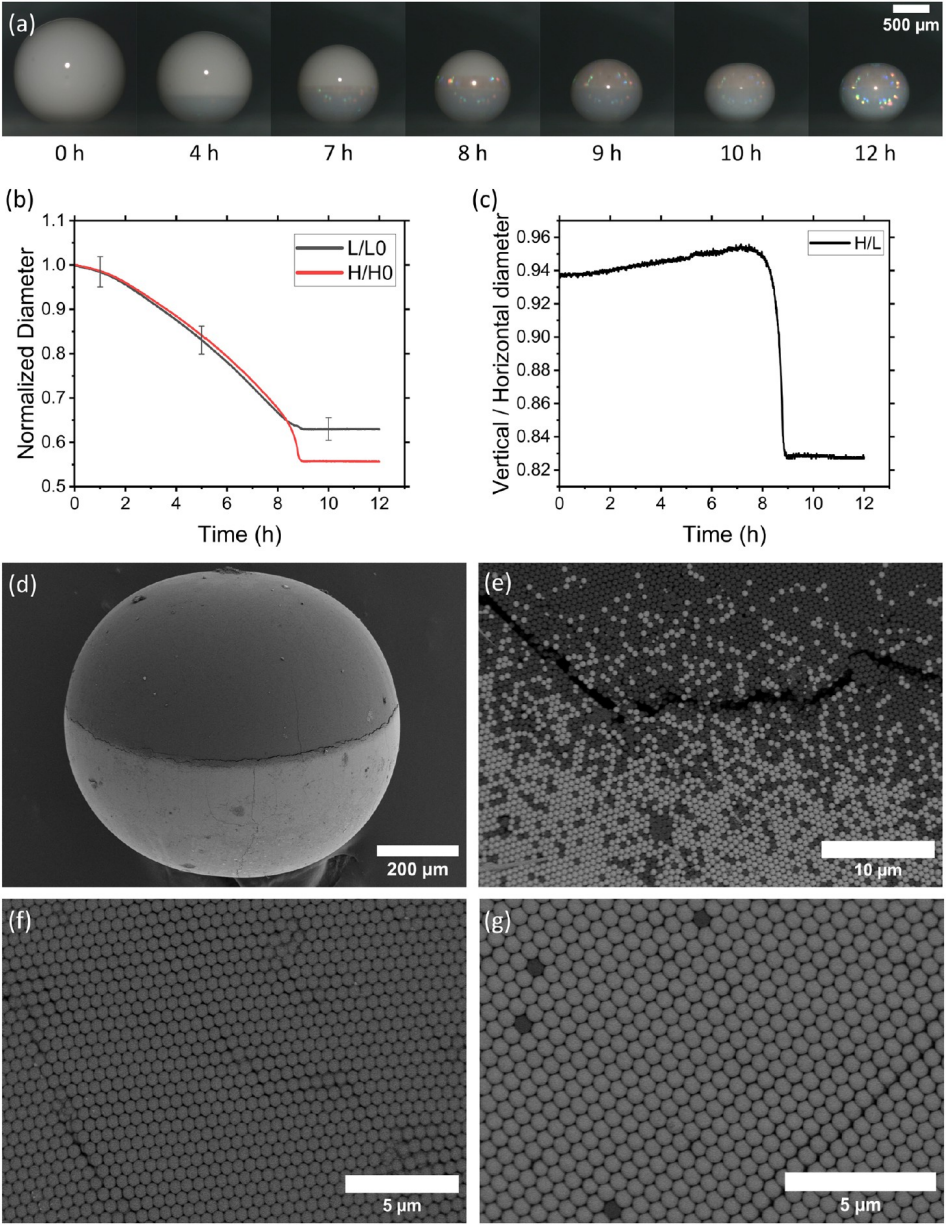
Formation of a Janus supraparticle by evaporation of an aqueous suspension containing a mixture of polystyrene (diameter, *d* = 608 nm) and silica (*d* = 622 nm) nanoparticles. (a) Evaporation of a binary suspension on a superamphiphobic surface. (b) Reduction of vertical (H) and horizontal diameter (L) over time, normalized with initial vertical (H0) and horizontal (L0) diameters during evaporation. (c) Change in ratio between vertical and horizontal diameter (H/L) over time. SEM images of (d) the final supraparticle with polystyrene on top and silica at the bottom, (e) PS-silica boundary, (f) polystyrene phase from top, and (g) silica phase from bottom.

The strong negative surface charge stabilizes the suspension during the early stages of drying by preventing aggregation. During evaporation, the reduction of solvent increases the particle volume fraction and raises the ionic concentration in the remaining liquid. This leads to compression of the electrical double layer and a reduction of the Debye length, which weakens electrostatic repulsion and allows particles to approach sufficiently closely for dense packing and crystallization. However, if the ionic strength is increased too much, particle aggregation can happen before crystalline ordering is achieved. Therefore, controlled addition of salt provides a means to tune the packing structure that varies from crystalline to amorphous structure.^60^

We monitored the change in vertical and horizontal diameters of the droplet with time. The droplet was nearly spherical at the beginning, with the horizontal diameter (L) slightly greater than the vertical diameter (H). This variation reduces while drying. The change in the ratio between the vertical and horizontal diameters suggests that the droplet approaches a more precise spherical shape while drying. This is expected when a sessile droplet dries on a superamphiphobic surface, as explained in previous works.^11,20^ However, in the final stage of drying, particle packing of the settled SiO_2_ makes the bottom hemisphere more viscous and solidifies earlier than the top hemisphere with dispersed PS. The position of the air–water interfaces is pinned in the lower part by the capillary forces keeping the three-phase contact line at the position of the outer particle layer, effectively fixing the shape of the lower part. Evaporation continues throughout the droplet, but the main change happens in the top part. Here, mobility is still high enough to allow deformation by the advection of the air–water interface. This asymmetry in deformability leads to a rapid reduction in vertical diameter (Figure 3b,c). Thus, the final structure is not a perfect sphere. We then recovered the SP by slightly tilting the substrate. Furthermore, phase separation is verified with SEM images (Figure 3d,e). The contrast difference indicates the phase separation of the particles. The brighter bottom hemisphere is SiO_2_, and the darker top hemisphere is PS. Both PS and SiO_2_ are arranged in an entropically favorable hexagonal close packing (hcp) with a sharp boundary (Figure 3f,g). We also observe a few counter particles trapped on either side. The uneven drying of the top and bottom hemispheres in the final stage creates mechanical stress at the boundary between PS and SiO_2_. As a result, a crack develops at the interface. For further analysis of the distribution of particles inside SP, we cut the SP in half (with a WEDO leather knife). We confirmed the phase separation on the inside using the SEM image from the interior of the particle (Figure 4a,b). Analysis of cross-sectional SEM images shows that the internal packing is not single-crystalline. Instead, both PS and SiO_2_ domains consist of polycrystalline assemblies composed of locally ordered regions exhibiting hcp and cubic close packing (ccp), together with a limited amorphous fraction (Figure 4c,d). To evaluate the degree of ordering, we performed a Fast Fourier transform (FFT) analysis of SEM images obtained from cross sections. The FFT patterns display diffuse ring features with discrete intensity maxima, indicating ordered arrangement (Figure S2). The analysis shows comparable polycrystalline ordering in both particle domains with a limited amorphous region, suggesting that NPs solidify at the air–water interface while solvent remains trapped inside during the final stage of evaporation. The confined interior limits particle rearrangement as drying proceeds, resulting in the presence of amorphous regions, alongside polycrystalline domains.

**Figure 4:**
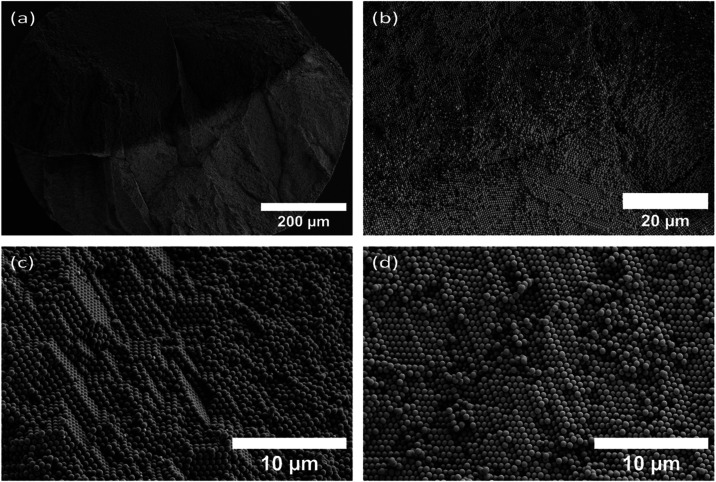
SEM image from the interior of Janus supraparticle containing a mixture of polystyrene (diameter, *d* = 608 nm) and silica (*d* = 622 nm) nanoparticles (RH > 95%). (a) Overview image of supraparticle after cutting, (b) PS-silica boundary, (c) polystyrene phase, and (d) silica phase.

### Sedimentation Inside Droplet

To better understand the balance between sedimentation and evaporation speed, evaporation of droplets with single components was tested along with binary mixtures. We chose PS NPs of mean diameters 608 nm (labeled with the fluorescent dye Nile red) and 1.8 μm. Similarly, we chose smaller and larger SiO_2_ with mean diameters of 152 and 622 nm. Two microliters of NP suspensions were dispensed onto the superamphiphobic surface at 97% RH. The image of the droplet at the beginning (Figure 5a–d) and after 4 h of evaporation (Figure 5e–h), before the complete drying, is captured. At the beginning of evaporation, NPs were homogeneously dispersed all over the droplet (Figure 5a–d). After 4 h, PS spheres with a mean diameter of 608 nm and SiO_2_ spheres with a mean diameter of 152 nm are still dispersed all over the droplet (Figure 5e,g). SiO_2_ spheres pack closely, leaving few internal interfaces, making them less scattering, resulting in a transparent droplet (dark upper hemisphere in Figure 5g, indicating the reflection of the surface). PS spheres with a mean diameter of 1.8 μm and SiO_2_ spheres with a mean diameter of 622 nm sediment after 4 h (Figure 5f,h), leaving a transparent water layer on top (the dark part in the upper hemisphere of the droplet in Figure 5f,h comes from the reflection of the surface). This clearly demonstrates NP sedimentation inside a droplet for higher buoyant masses before complete evaporation.

**Figure 5:**
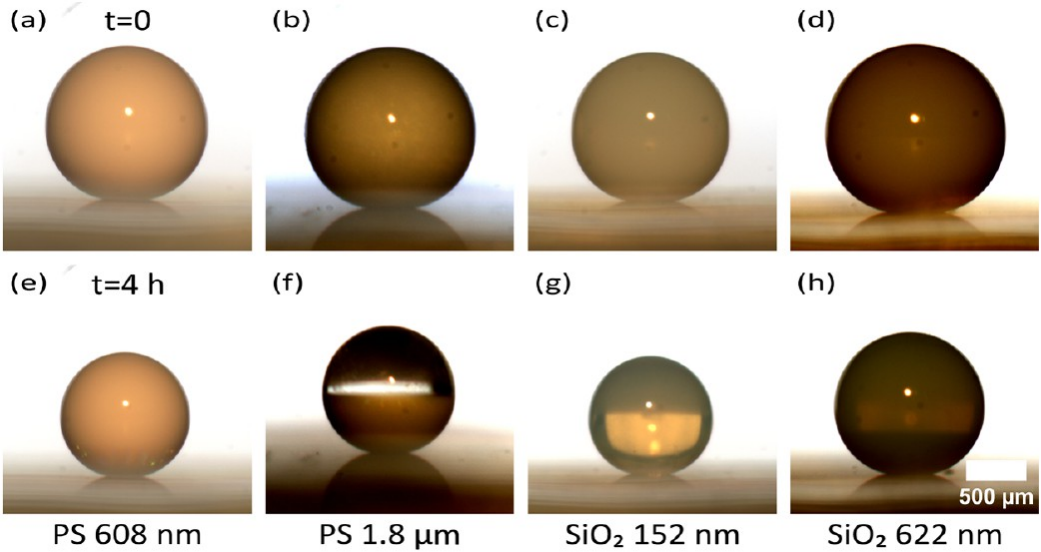
Sedimentation behavior of nanoparticles inside an evaporating droplet. Side-view image of evaporating droplets with polystyrene spheres of average diameters (a, e) 608 nm, (b, f) 1.8 μm, and silica spheres with average diameters (c, g) 152 nm and (d, h) 622 nm at the starting of evaporation (a–d) and after 4 h (e–h) at 97% relative humidity. The small PS (608 nm) and silica (152 nm) remain well-dispersed after 4 h, whereas the larger PS and silica particles show pronounced sedimentation.

### Supraparticles from Particles with Different Sizes

To further explore the influence of particle size on the fabrication process, we prepared a binary mixture using PS spheres of mean diameter 1.8 μm and SiO_2_ NPs of mean diameter 152 nm, following the same fabrication protocol as before. This combination consists of PS particles that settle faster in a droplet with individual species and SiO_2_ particles that diffuse in a droplet without settling (Figure 5) in an individual species system. However, in the binary mixture, the expected phase separation was reversed. After drying, we recovered the JSP with SiO_2_ at the bottom and PS at the top. This reversal suggests that, in multicomponent systems, density effects can become more dominant, as discussed in detail by Spruijt et al.^56^ Although SiO_2_ settled, the phase separation was not complete. Cross-sectional images show that a part of the SiO_2_ NPs remains within a mixed PS-SiO_2_ region. The final structure consisted of a pure SiO_2_ bottom hemisphere and a mixed PS–SiO_2_ top hemisphere. Notably, the boundary between the two regions was not sharp but showed a gradual transition from a higher to a lower SiO_2_ concentration (Figure 6a) from the bottom to the top.

**Figure 6:**
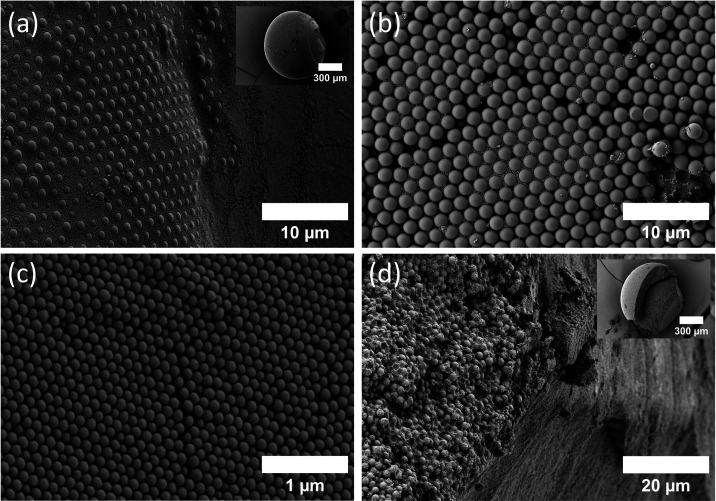
Janus supraparticle formed from nanoparticles with different sizes by evaporation of an aqueous suspension containing a mixture of polystyrene (*d* = 1.8 μm) and silica (*d* = 152 nm). (a) The boundary between pure silica and PS-silica mixture phase on the surface, the inset shows the whole Janus supraparticle, (b) PS-silica mixed phase from top hemisphere, (c) pure silica phase from bottom hemisphere, and (d) boundary between pure silica and PS-silica mixture phase from inside; the inset shows the particle after cutting.

With this combination of particles, effects such as crystallization and entropy become more evident with gravity. The fraction of SiO_2_ that is diffused throughout the droplet assembles together with PS at the top. To maximize entropy, the smaller SiO_2_ particles fill the voids between the assembled, crystallized PS particles (Figure 6b). At the bottom, phase-separated SiO_2_ crystallizes, leading to the removal of larger PS particles from their lattice sites. This results in a pure SiO_2_ phase at the bottom (Figure 6c). To examine the internal structure, we cut the SP. The interior showed a particle assembly and a gradient boundary similar to that observed on the surface (Figure 6d).

### Supraparticles from Particles with the Same Density

Up to now, we have demonstrated phase separation for particles with different densities. According to eqs 1 and 2, sedimentation can also be controlled by particle size. To further explore the influence of particle size on sedimentation and phase separation, we used a binary mixture of particles of the same material but with different sizes. We prepared a binary mixture of PS spheres of mean diameters of 1.8 μm and 608 nm as primary particles. We followed the same fabrication protocols. This system allows us to observe the combined influence of entropy, crystallization, and gravity. The sedimentation-dominated large PS settles, while the diffusion-dominated small PS remains diffused throughout the droplet. To maximize entropy, the smaller particles fill the voids of the larger PS crystals, disrupting the order and driving the system from a crystalline arrangement toward an amorphous structure at the bottom. On the top hemisphere, small PS forms crystals, and the larger particles are pushed out due to the combined effects of crystallization and gravity. As a result, the final SP shows a pure small-PS region on the top hemisphere and a mixed region at the bottom (Figure 7a,b). The boundary between the regions is a gradual change in concentration from large to small PS­(Figures 7c and S3). This gradual transition also avoids mechanical stress, preventing crack formation. To confirm the internal arrangement, we cut and imaged the SP, showing the same gradient structure inside (Figures 7d and S3). This approach shows how using particles of different sizes, but the same material, can reverse the internal structure compared to PS–silica mixtures with different sizes and gives a controlled way to create Janus structures with tunable internal composition.

**Figure 7:**
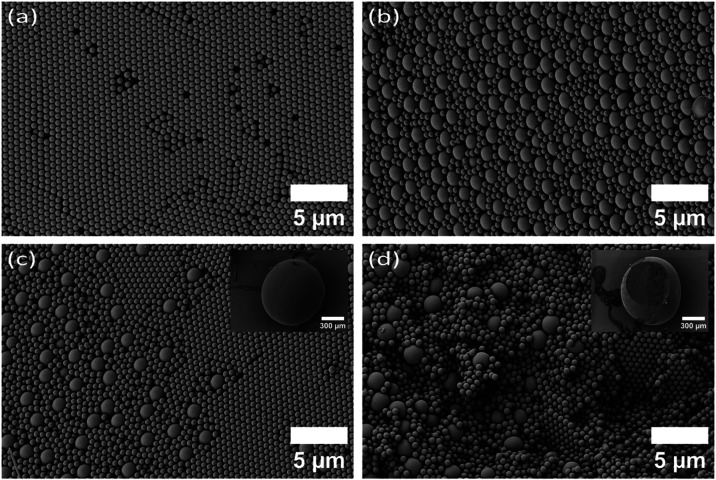
Janus supraparticle formed from particles with the same density by evaporation of an aqueous suspension containing a mixture of polystyrene nanoparticles of diameter 608 nm and 1.8 μm. (a) Pure crystalline polystyrene phase (*d* = 608 nm) from the top hemisphere and (b) amorphous polystyrene mixture phase from the bottom hemisphere. (c) Boundary between pure smaller polystyrene phase and polystyrene mixture phase from the surface with the whole supraparticle in the inset, and (d) phase boundary from inside with the whole supraparticle after cutting in the inset.

### Enhancing the Stability

To further understand how the drying process influences structural stability in JSPs, we explored the role of boundary sharpness and the drying rate. In previous experiments with particles with the same size but different densities, SPs are formed with pure phases on either hemisphere and a sharp phase boundary. However, this sharp interface led to mechanical stress during the final stage of drying, resulting in crack formation. In contrast, when we used PS particles of different sizes, we observed a gradient boundary and no cracks (Figure 7c). This suggests that a gradient change in the phase boundary improves the structural stability of SP compared to a sharp shift.

Building on this, we tested whether the drying speed could also control boundary sharpness and avoid crack formation. We prepared a binary mixture of PS NPs of mean diameter 608 nm and SiO_2_ NPs with a mean diameter of 622 nm. Then, drying of the droplet containing this mixture was observed under precisely controlled RH. By maintaining the RH in the range of 91%–93%, we reduced the drying time to the range of 4–5 h (Figure 8a). Under these conditions, we obtained a Janus structure with a gradient boundary from PS to SiO_2_ without cracks at the interface (Figures 8c and S4). However, further increasing the drying speed reduced phase separation, ultimately leading to no phase separation. This systematic variation of the RH revealed distinct structural regimes. At RH above 95%, droplets result in SPs with a sharp boundary. At RH 91–93%, a gradient interface was observed. Below 91% RH, phase separation was not observed, consistent with shorter evaporation times that limit sedimentation.

**Figure 8:**
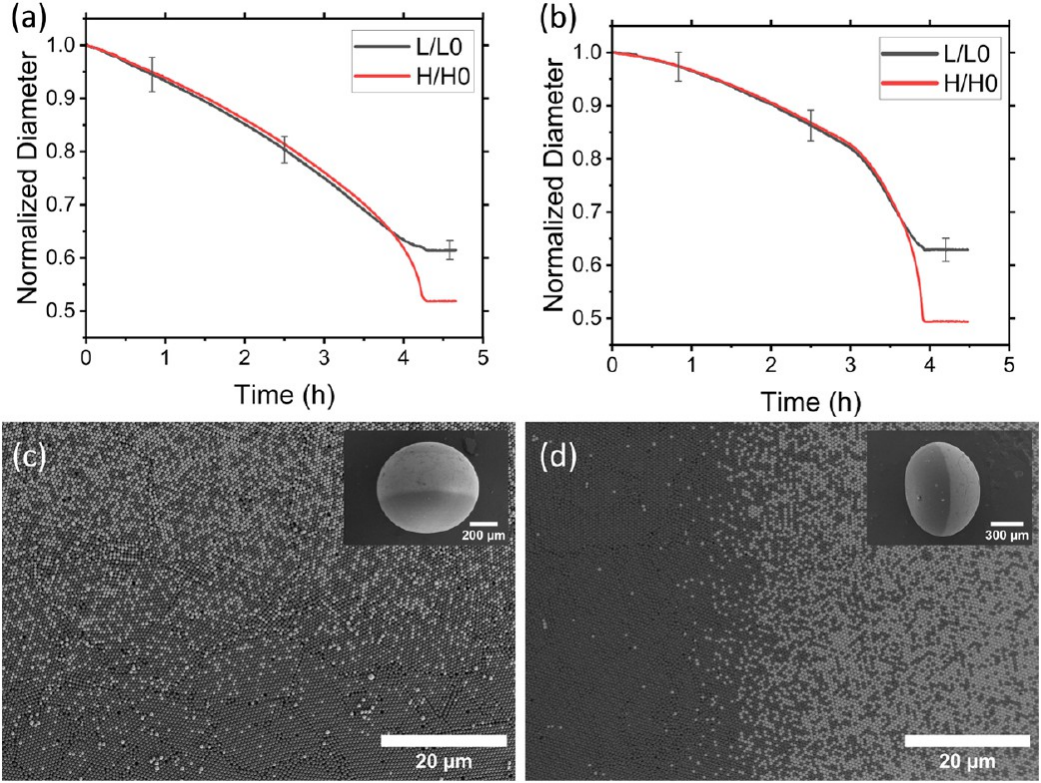
Formation of a Janus supraparticle with enhanced stability by fast evaporation of an aqueous suspension containing a mixture of polystyrene (*d* = 608 nm) and silica (*d* = 622 nm) nanoparticles. Fast evaporation results in a pronounced Janus structure with a gradient phase boundary while suppressing crack formation. (a) Reduction of vertical (H) and horizontal diameter (L) of the droplet, normalized with initial vertical (H0) and horizontal diameter (L0) at precisely controlled constant evaporation rate (RH = 91–93%), (c) resulted in a gradient boundary between polystyrene and silica phase with the whole Janus supraparticle in the inset. (b) Reduction of normalized vertical and horizontal diameter of droplet at varying 2-step evaporation rate, (d) resulted in a gradient boundary between polystyrene and silica phase with the whole Janus supraparticle in the inset.

Furthermore, we developed an easy-to-control two-step drying method to improve the stability. The primary particles chosen are the same. In the first stage, we kept the droplet in a high humid environment with an RH above 95% for 3 h to allow sedimentation. In the second stage, we gradually reduced and maintained the RH in the range of 85%–90% until the droplet dried (Figure 8b). With this method, we complete the process within 5 h. Here, the change in RH is a continuous reduction, not a sudden shift. With this varying evaporation rate, the boundary exhibits a gradual change from PS to SiO_2_ without any cracks in the interface (Figures 8d and S5). This method offers a reproducible way to improve structural stability through controlled drying kinetics, while also reducing the required drying time.

We performed nanoindentation experiments on JSPs with sharp and gradient boundaries to check their mechanical robustness. Elastic modulus and hardness were measured. Indentations were made on a contact area of about 25 μm^2^ at the SiO_2_–PS boundary. We chose around a 1 μm indentation depth by using an indentation force of 1 mN. We could see the change in mechanical properties when moving from one phase to the other in modulus, hardness, and indentation depth. We could not identify any significant difference between JSPs with sharp and with gradient boundaries in these experiments. This is due to both the large scatter of the data in the PS part of SP and the limited lateral resolution of the method (distance between indents had to be larger than the typical indentation footprint of roughly 25 μm^2^, for details see Figure S6). We obtained the Young’s modulus in the order of hundreds of M*Pa* to a few G*Pa* and hardness values of tens of MPa in both domains (Figure S6). This demonstrates sufficient mechanical stability for handling and storage of the SPs.

## Conclusion

We demonstrate a simple approach to fabricate spherical Janus supraparticles from colloidal binary mixtures based on the evaporation of droplets on superamphiphobic surfaces. Phase separation is induced by gravity. The sedimentation time of the particles prior to evaporation is controlled by humidity. To achieve different sedimentation times, we prepared binary mixtures of particles with varying densities and sizes. Droplets of mixtures containing particles with similar sizes but different densities resulted in Janus supraparticles with pure domains and sharp phase boundaries, whereas mixtures with different particle sizes produced at least one mixed domain with a gradient boundary. Notably, supraparticles composed of one pure domain and one mixed domain exhibit reduced interfacial stress during evaporation, thereby preventing crack formation at the boundary. Based on this idea, we are able to create more stable Janus supraparticles without any cracks, having pure domains and gradient phase boundaries either by precisely controlling the relative humidity or by employing a simple two-step process by varying humidity. Furthermore, other binary mixtures with nanoparticles of varied densities and sizes can be utilized to tailor the formation of mixed or pure domains, depending on the intended application. This study demonstrates that spherical Janus supraparticles can be fabricated through a simple, one-step process governed solely by gravity, a fundamental natural force.

## Experimental Methods

### Materials

Methyl trichlorosilane (TCMS, 99%, Sigma-Aldrich), hexadecane (Reagent Plus, 99%, Sigma-Aldrich), and 1*H*,1*H*,2*H*,2*H*-perfluorodecyltrichlorosilane (PFDTS, 96%, Alfa Aesar) were used in the preparation of superamphiphobic surfaces. Sodium metatungstate monohydrate (Alfa Aesar) was used to measure the buoyant density of NPs. N-hexane (≥95%) and toluene (≥99.8%) were purchased from Fisher Chemical. Acetone (≥99.8%) and ethanol (≥99.8%) were provided by Honeywell Research Chemicals. Ultrapure water with a resistivity of 18MΩ·cm was obtained by using a Sartorius Arium 611 VF water purification system. Glass slides, 25 × 75 mm^2^ in size, were provided by Menzel-Gläser, Germany. PS colloids (with −COOH groups on the surface) of diameter 608 ± 8.8 nm with a buoyant density of 1060 kg/m^3^ (ζ-potential = −43 mV, with Nile red fluorescent dye) and 1793 nm ± 18 nm with a buoyant density of 1080 kg/m^3^ (ζ-potential = −41 mV) were synthesized by the copolymerization of styrene and acrylic acid using surfactant-free emulsion polymerization.^61^ SiO_2_ particles of diameter 152 nm ± 1.5 nm with a buoyant density of 1890 kg/m^3^ (ζ-potential = −54 mV) and 622 ± 6.8 nm with a buoyant density of 1880 kg/m^3^ (ζ-potential = −57 mV) were synthesized by Stöber process.^62^ The synthesized colloids were purified by several centrifugation cycles and finally redispersed in ultrapure water.

### Preparation of Superamphiphobic Surfaces

The silicon nanofilament-based superamphiphobic surfaces were prepared as described previously.^57,58^ Glass slides were cleaned by ultrasonicating with toluene, ethanol, and acetone and dried by keeping in an oven for 1 h at 60 °C. Then, glass slides were oxygen plasma-treated at 80 W for 2 min (Diener Electronic Femto). We prepared a mixture of 100 mL toluene with 150–170 ppm water content and added 0.4 mL of trichloromethylsilane (TCMS). Then, we immersed the activated glass slides in the solution for 5 h. After the reaction, the silicone nanofilament-grafted glass slides were rinsed with hexane to remove unreacted TCMS and then dried by keeping them in an oven for 1 h at 60 °C. In order to fluorinate the substrate, the nanofilaments were again activated by oxygen plasma at 80 W for 2 min. A mixture of 100 mL of hexane and 100 μL of trichloroperfluorodecylsilane is prepared. Activated glass slides were immersed in this mixture for 1 h. The substrates were rinsed with hexane to remove any unreacted fluorosilanes. The static contact angle of the obtained glass slides is 157° for water droplets and 148° for hexadecane droplets at a volume of 6 μL.

### Evaporation of Colloidal Suspension on Superamphiphobic Surfaces

The aqueous suspensions were prepared prior to the experiments. We used SiO_2_ NPs with a mean diameter of 622 and 152 nm at a volume fraction of 12% and PS particles with a mean diameter of 608 and 1793 nm with 15% and 10% volume fraction, respectively. With these primary NPs, 3 binary mixtures were prepared: PS/SiO_2_ with particles of similar size, PS/PS with different sizes, and larger PS with smaller SiO_2_. Two microliters of each primary particle suspension and mixtures were dispensed on the superamphiphobic surface. The drying was conducted in a humidity chamber built in-house with an inlet for nitrogen gas flow to control humidity and a window through which the evaporation process was monitored by a camera (Blackfly S BFS-U3–50S5C). Droplets are illuminated from the same direction as the camera using a lamp with an optic fiber waveguide to observe the phase separation during evaporation. Additionally, an LED backlight (Aputure MC) is used to improve the contrast of the drop boundary for characterization of the overall droplet shape. The RH was controlled by mixing a flow of nitrogen that was saturated before by letting it bubble through water and dry nitrogen. The Nitrogen flow through water is set in the range of 400–500 standard cubic centimeters per minute (SCCM). The dry nitrogen flow is set at zero and is used only for final precision. For the experiments with two different ranges of RH, we initially saturated the chamber at 400–500 SCCM and after 3 h maintained the flow at 0–50 SCCM. The RH during evaporation of the suspension drops was recorded with the help of a humidity sensor inside the chamber. Once the desired RH was reached, the droplets were dispensed through the holes on top of the chamber, and the holes remained closed throughout the evaporation. The suspension droplets evaporated at 22 °C in a range of relative humidity from 85% to 99%. High RH above 95% is achieved by placing one or two wet tissue papers inside the humidity chamber. The supraparticles formed after evaporation were released by slightly inclining the surfaces and were collected in small vials.

### Characterization

Static contact angles of 6 μL of water and hexadecane droplets on a superamphiphobic surface were measured by a goniometer, Krüss Advance (Krüss GmbH, Germany), using an elliptic fit of drop shape. Buoyant densities of NPs were determined by density matching in aqueous sodium metatungstate monohydrate (SMT) solutions, where the SMT concentration was adjusted until the particles remained suspended without floating. The morphologies of the superamphiphobic surfaces and the supraparticles were imaged by SEM (ZEISS GeminiSEM 560, Germany). Prior to imaging, the samples were sputter-coated with a 14 nm layer of platinum using a Safematic Compact Coating Unit-010 to avoid charging. Coating is done in 2 steps of 7 nm by tilting the sample 15° and 35° on each step to ensure sufficient coating toward the contact point of supraparticles on the substrate. The supraparticles composed of PS 608 nm mixed with SiO_2_ 622 nm were not coated with platinum in order to obtain effective material contrast since their size range is the same. The size and polydispersity of the particles were determined by dynamic light scattering (Malvern Zetasizer Nano S90). The ζ-potentials of the particles were measured by a zetasizer (Malvern Zetasizer Nano Z). Measurements were performed at pH between 5.5 and 6 without the addition of salt. We performed nanoindentation experiments using an MFP3D AFM from Asylum Research with nanoindentation head. As a probe, we used a diamond Berkovich indenter. To ensure stable mounting of the samples, we placed the SP on a glass slide that had been blade-coated with a very thin layer of two-component epoxy glue (UHU Endfest, UHU GmbH, Germany). Using ImageJ software, we performed image analysis of the evaporation videos of the droplets and recorded the change in droplet’s vertical and horizontal diameters as a function of time. The diameter is determined by edge detection.

## Supplementary Material





## Notes

Formation of JSP from an aqueous suspension of PS–SiO_2_ mixture (Video S1) (MP4)
Images of the superamphiphobic surface (Figure S1); FFT analysis confirming structural ordering within the SP (Figure S2); additional SEM images illustrating SPs formed with PS particles of different sizes (Figure S3); complementary SEM images providing magnified and internal views of the JSP composed of PS–SiO_2_ with enhanced stability corresponding to those shown in the main text (Figures S4 and S5); and a brief description of the nanoindentation measurement used to assess their mechanical properties (Figure S6) (PDF)
