# Supplementary material for: Fabrication of Janus Supraparticles by Induced Phase Separation by Gravity
Source: ACS Nano. 2026 Apr 15;20(16):12285–95. doi: 10.1021/acsnano.5c20500 (PMC13131045; doi:10.1021/acsnano.5c20500)
Supplement: Supplementary file 2 [file nn5c20500_si_002.pdf]

Supporting Information: Fabrication of Janus Supraparticles by Induced Phase Separation by Gravity

Sayanth Ramachandran,† Michael Kappl,*,† Marcel Sperling,‡ Michael Gradzielski,‡ and Hans-Jürgen Butt†

†*Department of Physics at Interfaces, Max-Planck Institute for Polymer Research, 55128 Mainz, Germany*

‡*Stranski-Laboratorium für Physikalische und Theoretische Chemie, Technische Universität Berlin, Strasse des 17. Juni 124, Berlin 10623, Germany*

E-mail: kappl@mpip-mainz.mpg.de

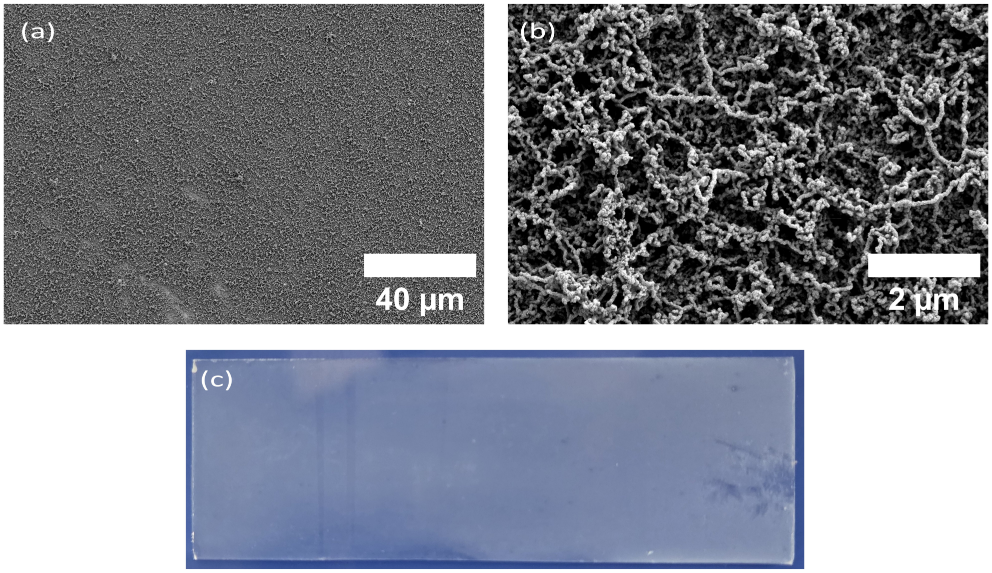

Figure S1: (a) zoom-out and (b) zoom-in SEM images showing the surface morphology of silicone nanofilament-coated glass substrates and (c) photo of the substrate.

- Video S1: Drying video of the formation of a Janus supraparticle by evaporation of an aqueous suspension containing a mixture of polystyrene (diameter, $d = 608$ nm) and silica ($d = 622$ nm) nanoparticles (playback speed 5× relative to real time)

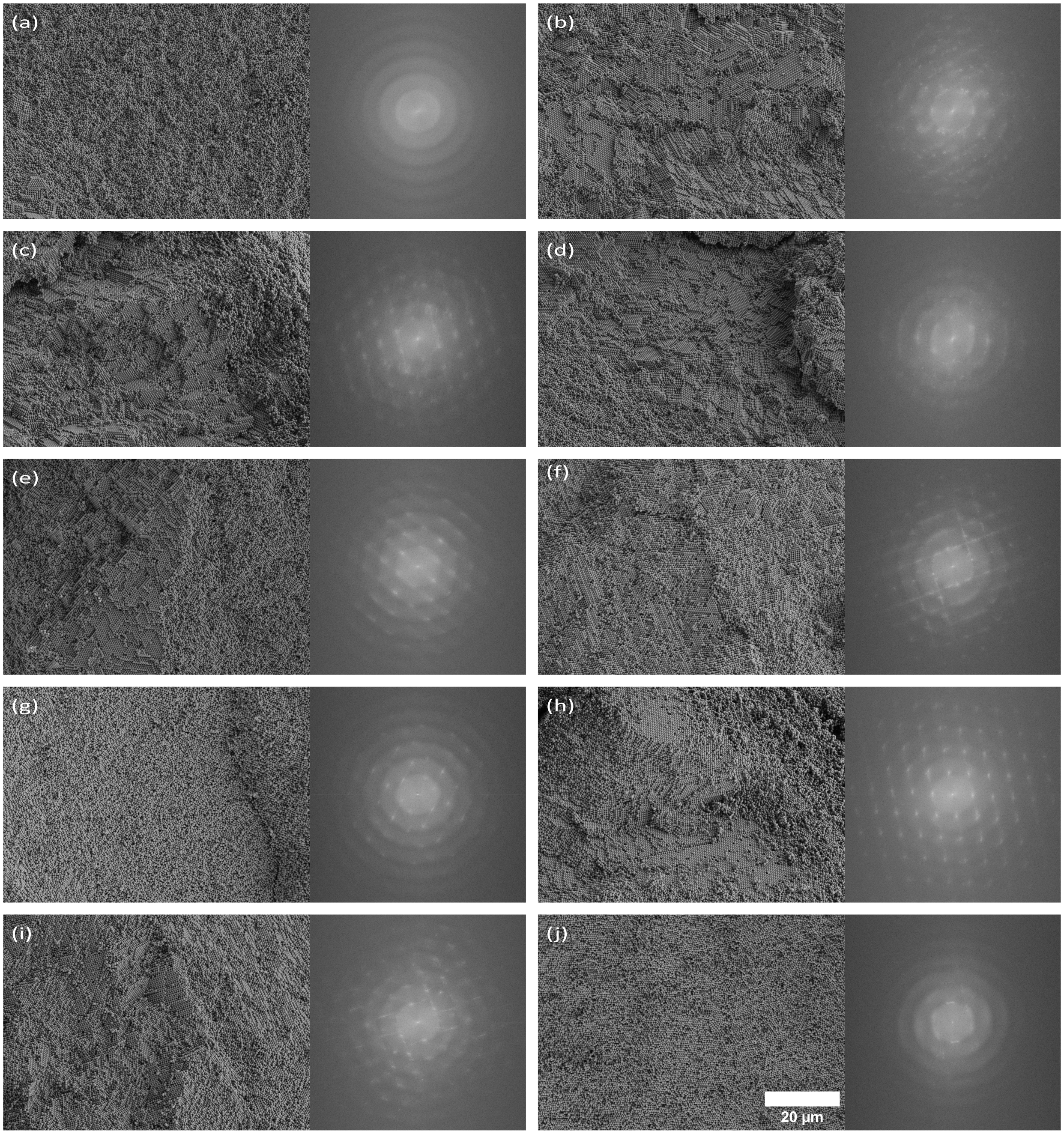

Figure S2: SEM image and Fast Fourier transform (FFT) analysis from the interior of Janus supraparticle containing a mixture of polystyrene (diameter, $d = 608$ nm) and silica ($d = 622$ nm) nanoparticles (RH $>$ 95%). (a-e) Different regions on polystyrene phase, (f-j) Different regions on silica phase.

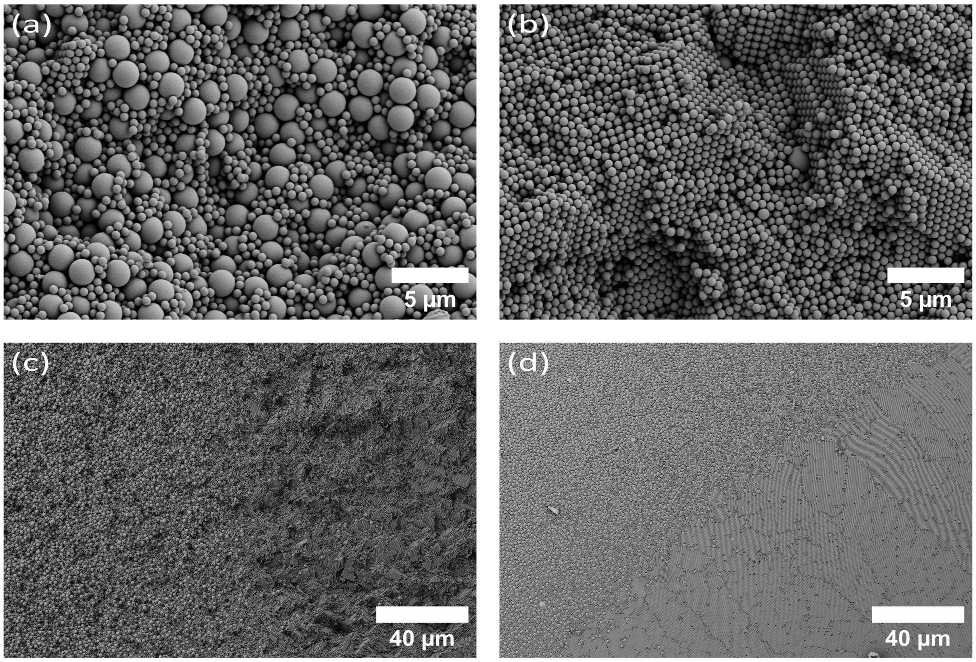

Figure S3: SEM image of Janus supraparticle obtained after evaporation of an aqueous suspension containing a mixture of polystyrene nanoparticles of diameter 608 nm and 1.8 μm. (a) smaller-larger mixed phase of polystyrene on bottom hemisphere and (b) pure smaller polystyrene ($d = 608$ nm) phase on top hemisphere from inside. Overview image of boundary between mixed phase and pure phase (c) from inside and (d) from surface.

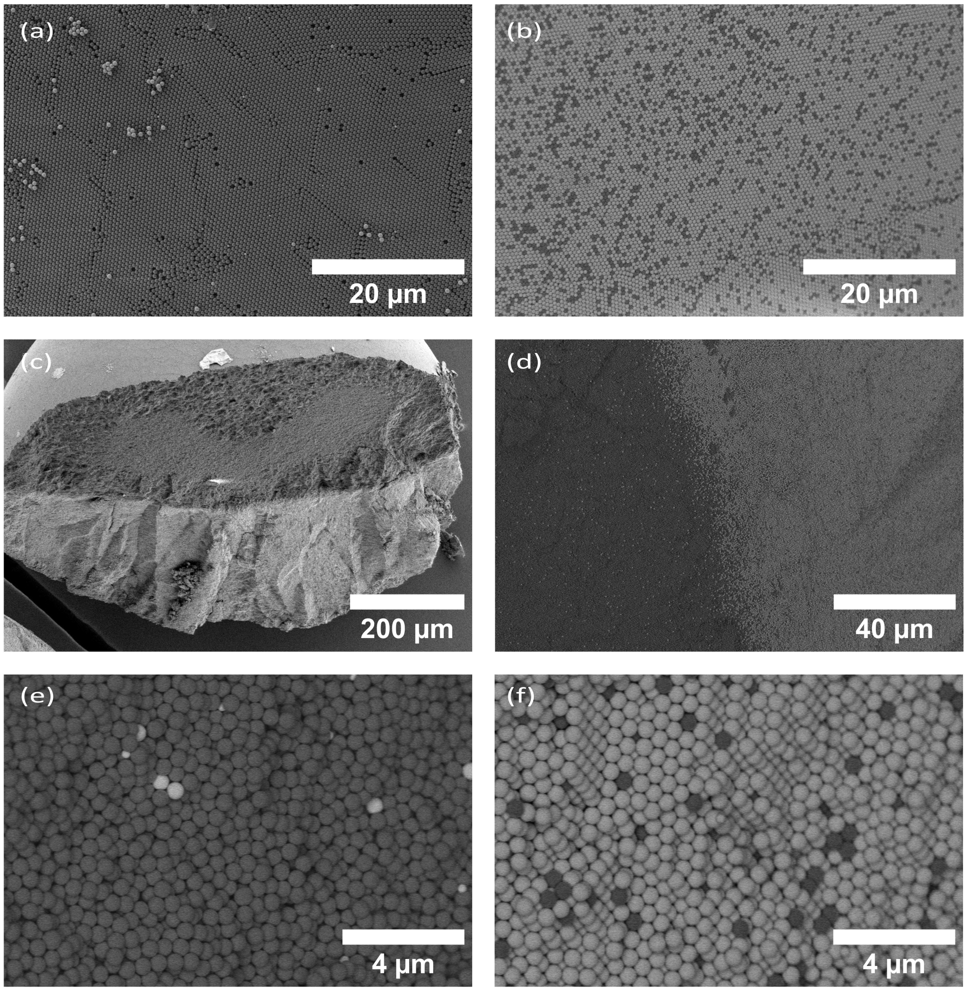

Figure S4: SEM image of Janus supraparticle by evaporation of an aqueous suspension containing a mixture of polystyrene (diameter, d = 608 nm) and silica (d = 622 nm) nanoparticles with a precisely controlled evaporation rate (RH= 91%-93%). This increased evaporation rate results in a pronounced Janus structure with a gradient phase boundary while suppressing crack formation.(a) Polystyrene phase on top hemisphere and (b) silica phase on bottom hemisphere at surface, (c) interior structure of Janus supraparticle after cutting, (d) PS-silica boundary from inside, (e) polystyrene phase and (f) silica phase from inside.

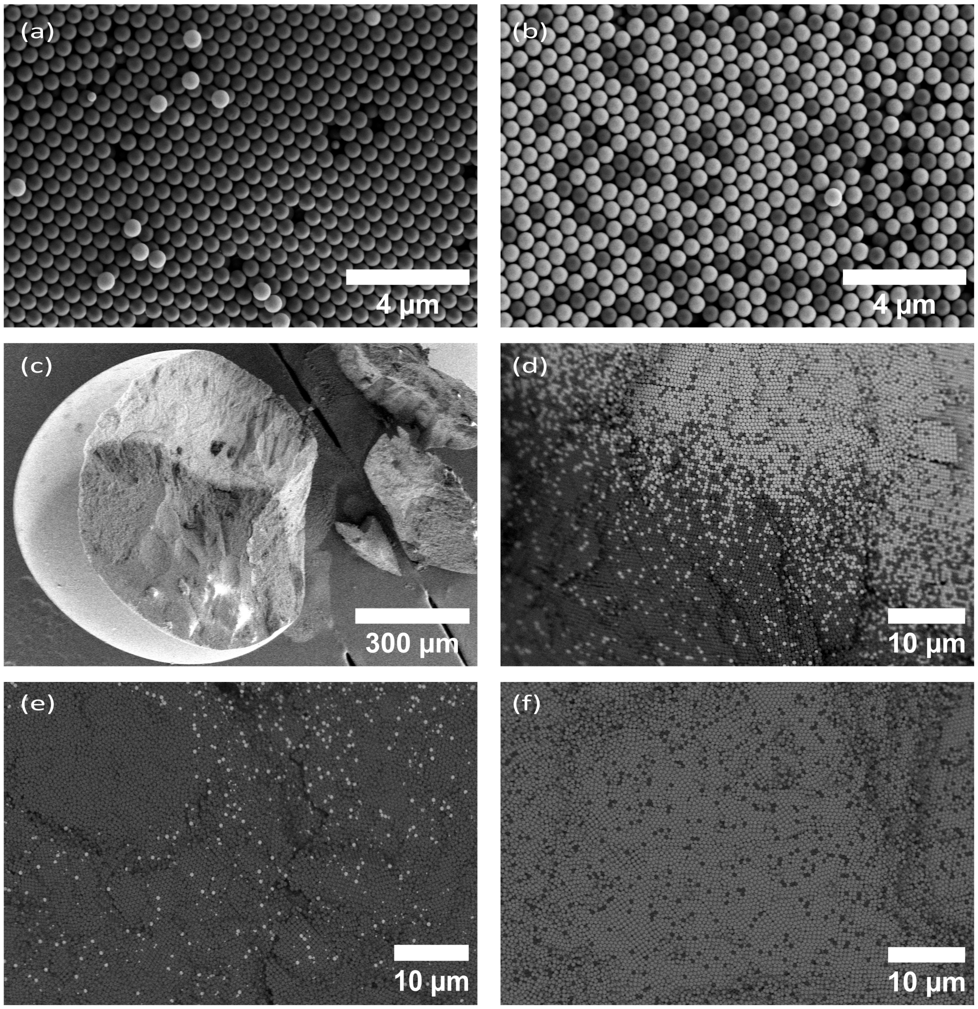

Figure S5: SEM image of Janus supraparticle by evaporation of an aqueous suspension containing a mixture of polystyrene (diameter, d = 608 nm) and silica (d = 622 nm) nanoparticles with a varying 2-step evaporation rate. Here drying time is reduced, resulting in a Janus structure with a gradient phase boundary while suppressing crack formation. (a) Polystyrene phase on top hemisphere, (b) silica phase on bottom hemisphere at surface, (c) interior structure of Janus supraparticle after cutting, (d) PS-silica boundary from inside, (e) polystyrnee phase and (f) silica phase from inside.

Nanoindentation of the supraparticles

Nanoindentation of the SPs was carried out using a MFP3D AFM from Asylum Research with nanoindenation head. As probe we used a diamond Berkovich indenter. To ensure stable mouting of the samples, we placed the SPs on a glass slide which had been blade coated with a very thin layer of two component epoxy glue (UHU Endfest, UHU GmbH, Germany) and oriented under an optical microscope to ensure that the phase boundary between SiO_2 and PS domains was running over the top side of the SP. After curing of the glue, the glass slides with particles were clamped onto the sample stage of the nanoindenter. The integrated side view optics of the nanoindenter was used to identify the orientation of the phase boundary. In order to probe not just single particle properties, we chose typical indentation depth of roughly 1 μm by using an indentation force of 1 mN. Since this corresponds to a contact area of about 25 μm^2, we had to chose a lateral spacing between the indentation points of about 5 μm to avoid overlapping of the indents. The maximum lateral movement by the piezo stage of the nanoindenter was 90 μm, so we could place 20 indents along a 90 μm line. Young's modulus, hardness, and indentation depth were obtained by fitting of the experimental data using the Oliver-Pharr-model[S1] that is part of the Asylum Research data analysis software (version 14.13.134). For both types of particles, we could see the change in mechanical properties when moving from one phase to the other in modulus, hardness, and indentation depth. However, due to the large scatter of the data on the PS part and the limited lateral resolution, we could not identify any significant difference between the two types of samples (Fig. S6).

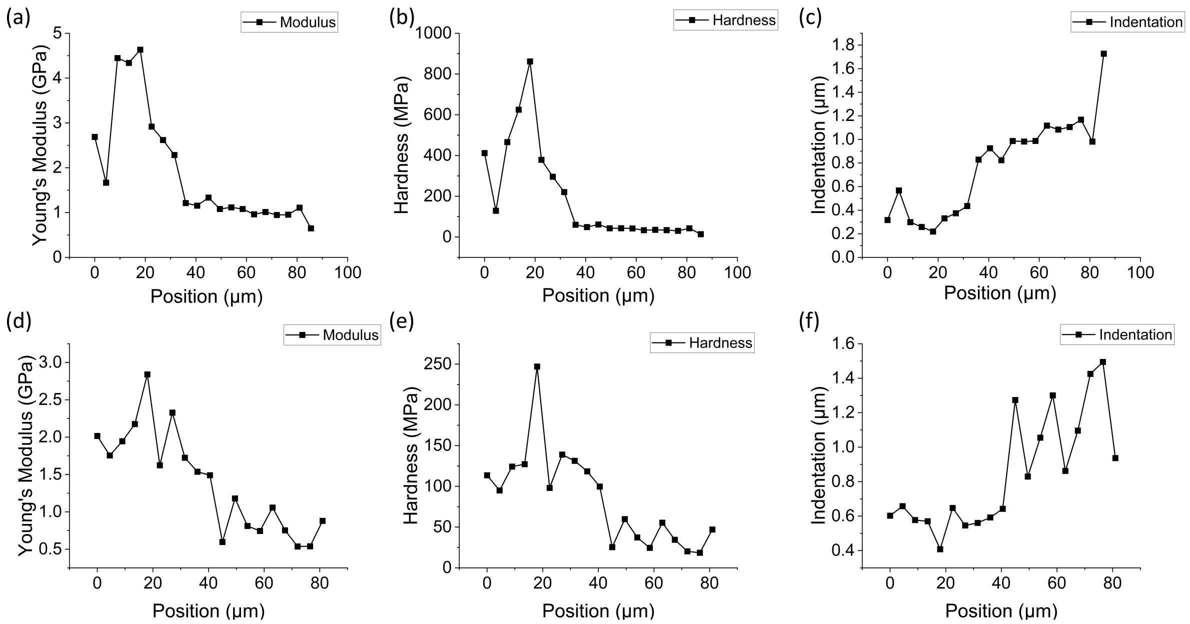

Figure S6: Nanoindentation of Supraparticles containing polystyrene (diameter, $d = 608$ nm) and silica ($d = 622$ nm). a) Young's modulus, b) Hardness, c) Indentation depth for a Supraparticle with gradient at phase boundary. d) Young's modulus, e) Hardness, f) Indentation depth for a Supraparticle with sharp phase boundary

References

(S1) Oliver, W. C.; Pharr, G. M. An improved technique for determining hardness and elastic modulus using load and displacement sensing indentation experiments. *Journal of materials research* **1992**, *7*, 1564–1583.
